# The role of point‐of‐care tests in antibiotic stewardship for urinary tract infections in a resource‐limited setting on the Thailand–Myanmar border

**DOI:** 10.1111/tmi.12541

**Published:** 2015-06-11

**Authors:** Lauren Chalmers, Jessica Cross, Cindy S. Chu, Aung Pyae Phyo, Margreet Trip, Clare Ling, Verena Carrara, Wanitda Watthanaworawit, Lily Keereecharoen, Borimas Hanboonkunupakarn, François Nosten, Rose McGready

**Affiliations:** ^1^ Shoklo Malaria Research UnitFaculty of Tropical MedicineMahidol UniversityMae SotThailand; ^2^ Mahidol‐Oxford Tropical Medicine Research UnitFaculty of Tropical MedicineMahidol UniversityBangkokThailand; ^3^ Centre for Tropical Medicine and Global HealthNuffield Department of Clinical MedicineUniversity of OxfordOxfordUK

**Keywords:** urinary tract infection, antibiotic resistance, extended‐spectrum *β*‐lactamase, point‐of‐care tests, infection des voies urinaires, résistance aux antibiotiques, BLSE, tests au point des soins

## Abstract

**Objective:**

Published literature from resource‐limited settings is infrequent, although urinary tract infections (UTI) are a common cause of outpatient presentation and antibiotic use. Point‐of‐care test (POCT) interpretation relates to antibiotic use and antibiotic resistance. We aimed to assess the diagnostic accuracy of POCT and their role in UTI antibiotic stewardship.

**Methods:**

One‐year retrospective analysis in three clinics on the Thailand–Myanmar border of non‐pregnant adults presenting with urinary symptoms. POCT (urine dipstick and microscopy) were compared to culture with significant growth classified as pure growth of a single organism >10^5^ CFU/ml.

**Results:**

In 247 patients, 82.6% female, the most common symptoms were dysuria (81.2%), suprapubic pain (67.8%) and urinary frequency (53.7%). After excluding contaminated samples, UTI was diagnosed in 52.4% (97/185); 71.1% (69/97) had a significant growth on culture, and >80% of these were *Escherichia coli* (20.9% produced extended‐spectrum *β*‐lactamase (ESBL)). Positive urine dipstick (leucocyte esterase ≥1 and/or nitrate positive) compared against positive microscopy (white blood cell >10/HPF, bacteria ≥1/HPF, epithelial cells <5/HPF) had a higher sensitivity (99% *vs*. 57%) but a lower specificity (47% *vs*. 89%), respectively. Combined POCT resulted in the best sensitivity (98%) and specificity (81%). Nearly one in ten patients received an antimicrobial to which the organism was not fully sensitive.

**Conclusion:**

One rapid, cost‐effective POCT was too inaccurate to be used alone by healthcare workers, impeding antibiotic stewardship in a high ESBL setting. Appropriate prescribing is improved with concurrent use and concordant results of urine dipstick and microscopy.

## Abstract (fr)

**Objectif:**

La littérature publiée provenant des régions à ressources limitées est rare, bien que les infections des voies urinaires (IVU) soient une cause fréquente de présentation ambulatoire et d'utilisation d'antibiotiques. L'interprétation des tests au point des soins (TPS) est liée à l'utilisation d'antibiotiques et à la résistance aux antibiotiques. Nous avons cherché à évaluer la précision diagnostique des TPS et leur rôle dans la prise en charge antibiotiques des IVU.

**Méthodes:**

Analyse rétrospective sur un an dans trois cliniques à la frontière entre la Thaïlande et le Myanmar sur des adultes non enceintes avec des symptômes urinaires. Les TPS (bandelettes urinaires et microscopie) ont été comparés à la culture avec une croissance significative, classée comme croissance pure d'un organisme unique >10^5^ cfu/mL.

**Résultats:**

Chez 247 patients, 82,6% de femmes, les symptômes les plus fréquents étaient la dysurie (81.2%), la douleur suspubienne (67.8%) et la fréquence urinaire (53.7%). Après exclusion des échantillons contaminés, les IVU ont été diagnostiquées chez 52.4% (97/185); 71.1% (69/97) ont révélé une croissance significative sur la culture et pour >80% d'entre eux, il s'agissait d’*Escherichia coli* (20.9% à spectre étendu de *β*‐lactamase (BLSE)). La positivité de la bandelette urinaire (estérase leucocytaire ≥1 et/ou de nitrate positive) comparée à la positivité de la microscopie (globule blanc >10/HPF, bactéries ≥1/HPF, cellules épithéliales <5/HPF), avait une sensibilité plus élevée (99% vs 57%), mais une plus faible spécificité (47% vs 89%). La combinaison des TPS menait à la meilleure sensibilité (98%) et spécificité (81%). Près d'un patient sur dix ont reçu un antimicrobien auquel l'organisme n’était pas totalement sensible.

**Conclusion:**

Un TPS rapide et rentable était trop imprécis pour être utilisé seul par les agents de la santé, ce qui entrave la prise en charge antibiotique dans un cadre à taux élevé de BLSE. Des TPS améliorés sont urgemment nécessaires pour faire face à cette résistance mondiale croissante.

## Abstract (es)

**Objetivo:**

Si bien las infecciones del tracto urinario (ITU) son una causa común de las consultas externas y del uso de antibióticos en lugares con recursos limitados, la literatura publicada al respecto es escasa. La intrepretación de las pruebas de diagnóstico en el punto de atención (PoC) se relaciona con el uso de los antibióticos y la resistencia a los mismos. Nuestro objetivo era evaluar la precisión diagnóstica de las PoC y su papel en la administración de antibióticos para ITU.

**Métodos:**

Análisis retrospectivo de un año en tres clínicas en la frontera entre Tailandia‐Myanmar para adultos no embarazados que con síntomas urinarios. Las pruebas diagnósticas PoC (tira reactiva de orina y microscopía) se compararon con el cultivo, clasificando un crecimiento significativo como un crecimiento puro de un único organismo si >10^5^cfu/mL.

**Resultados:**

En 247 pacientes, 82,6% mujeres, los síntomas más comunes eran disuria (81,2%), dolor suprapúbico (67,8%) y frecuencia urinaria (53,7%). Después de excluir las muestras contaminadas, se diagnosticó ITU en un 52,4% (97/185); 71,1% (69/97) tenían un crecimiento significativo en cultivo y >80% de ellos eran *Escherichia coli* (20,9% producían *β*‐lactamasas de amplio espectro (BLAEs)). Un resultado positivo con la tira reactiva de orina (esterasa leucocitaria ≥1 y/o nitrato positiva), comparado con una microscopía positiva (leucocitos >10/HPF, bacterias ≥1/HPF, células epiteliales <5/HPF), tenían una mayor sensibilidad (99% vs 57%) pero menor especificidad (47% vs 89%), respectivamente. Pruebas PoC combinadas tenían la mejor sensibilidad (98%) y especificidad (81%). Casi uno de diez pacientes recibió un antimicrobiano para el cual el patógeno no era totalmente susceptible.

**Conclusión:**

Una sola prueba PoC rápida y coste‐efectiva era demasiado inexacta para ser utilizada por si sola por los trabajadores sanitarios, dificultando la administración de antibióticos en zonas con una alta prevalencia de BLAEs. Se requiere con urgencia unas pruebas PoC mejoradas para abordar la cada vez mayor resistencia a antibióticos a nivel global.

## Introduction

In view of increasing antibiotic resistance in low‐income countries 1, 2, 3, efficient, economic and effective diagnostic and treatment strategies 4 are required for urinary tract infections (UTI), one of the most common reasons for adult outpatient attendance and antibiotic prescription 5. The WHO Antibiotic Resistance Surveillance 2014 report illustrated the presence of limited data in South‐East Asia and described the situation as a ‘burgeoning and often neglected problem’ 6, 7. In South‐East Asia, extended‐spectrum *β*‐lactamase (ESBL) rates are amongst the highest reported 8 and unrestricted antibiotic use and polypharmacy are rife 9. The roll out of rapid diagnostic tests (RDT) and artemisinin combination therapy for malaria have also revealed the millions of cases of fever misdiagnosed in tropical countries 10. Accurate discriminating diagnostic testing and structured diagnostic pathways for prescribing in non‐malaria fevers need to be developed 11.

A Cochrane review emphasises point‐of‐care tests (POCT) to allow informed antibiotic prescribing 12. Clinical features and POCT including urine dipstick and urine microscopy allow empiric treatment to be commenced before urine culture results are available. Overdiagnosis from this approach is accepted as delaying antibiotics by more than 48 h, whilst waiting for the culture results, is much more likely to have poor symptom control, emphasising the importance of obtaining a rapid decision 13.

History alone has a diagnostic sensitivity between 50% and 80%, and multiple algorithms used in different studies have made clinical assessment of UTI difficult to compare 14. Clinical examination is not useful in diagnosing UTI 15 as opposed to pyelonephritis. Urine dipstick is routine practice in many countries for initially investigating a UTI and a meta‐analysis (of 70 publications) concluded that a urine dipstick that was negative for both nitrites and leucocyte esterase was useful in all populations to exclude the presence of infection 16. The sensitivities of positive leucocyte esterase and/or nitrites ranged from 68% to 88% in different patient groups and indicated that positive results have to be confirmed 16. When positivity of urine dipstick was defined as the presence of nitrites or leucocyte esterase and blood, the sensitivity and specificity were 77% and 70% respectively and NPV 65% 17. Unfortunately, few conclusions have been drawn on the validity of urine microscopy. Its use seems to offer little added value to diagnosis based on history and dipstick 18. Urine culture for the detection and quantification of the pathogen and sensitivities from a mid‐stream urine (MSU) sample in the presence of clinical symptoms remains the UTI diagnostic gold standard 16.

The clinical decision‐making of primary healthcare workers in rural and remote areas is rarely assessed, particularly in regard to interpretation of the POCT 10, 19. As a remote example, due to paucity of published data, healthcare workers in Blantyre, Malawi correctly adhered to treatment of positive microscopy and RDT results for malaria, but also rarely withheld treatment in the setting of a negative result: 58% of patients with a negative RDT still received antimalarials 19. In fact, in rural and remote parts of Asia, the main uncertainty for malaria‐orientated primary healthcare workers is how to diagnose and treat non‐malaria febrile patients 11. An evaluation of prevalence in Panamanian women showed UTI was diagnosed in 29.8%, but only 21.15% met evidence‐based criteria, although this was not compared to urine culture 20.

Shoklo Malaria Research Unit (SMRU) has established clinics attended by remote populations scattered along the Thailand–Myanmar border that provide basic care but easy access to diagnosis and treatment of malaria by microscopy or rapid detection tests 21. Significant gains in control of malaria in the area and reductions in the numbers of positive cases have thrown the spotlight on diagnosis and treatment of non‐malaria diagnoses including UTI 22, 23.

In view of these issues, we aimed to conduct a one‐year retrospective analysis of non‐pregnant adult patients presenting with urinary symptoms to assess diagnostic accuracy of POCT, and their role in improving urinary tract infection antibiotic prescribing in remote clinics.

## Materials and methods

### Site and population

SMRU clinics are situated in Tak province, Thailand, adjacent to the Thailand–Myanmar border. There are two sites for migrants at Wangpha (WPA) and Mawker Thai (MKT) villages with a combined estimated population of 50 000–150 000 (mobile nature of the population makes it impossible to provide a single figure) and a further clinic in Maela Refugee Camp (MLA) with a population of 43 641 in June 2014 (www.theborderconsortium.org). Free health care to marginalised people from both sides of the border with outpatient and antenatal departments, and limited inpatient facilities are provided. The clinics’ case mix includes a variety of tropical infections, for example malaria, typhoid, rickettsial infections, dengue and other communicable and non‐communicable diseases. There is no schistosomiasis reported in this area. There are limited on‐site laboratory facilities which undertake microscopy for malaria blood smears, urine and faecal specimens. Since SMRU was established in 1986, urinary microscopy was, for many years, the only laboratory support for UTI diagnosis. Urine dipsticks became available in 2006 and urine culture in 2011.

Patients are able to access drugs through a variety of means. This includes non‐profit organisations such as SMRU, Thai public services (although payment and transportation via checkpoints is required), or in shops and pharmacies on both sides of the border, where villagers can purchase packets without medical assessment 9. These packets are known locally as *yaa chud* and consist of a mix of four to five tablets, of which 20% contain antibiotics according to mass and atomic spectrometry 9. Unpublished data from a survey suggested 25% of pregnant women had antimicrobial activity in their urine 24.

The healthcare providers are local staffs who have completed at least one‐year training to be competent at assessment and treatment of common diseases. In addition, they have support from qualified doctors.

Predominantly of Karen and Burman ethnicity, Carrara *et al*. reported a literacy rate amongst 2424 pregnant women of 47% in 2011 25. A glucose‐6‐phosphate dehydrogenase deficiency (G6PD) prevalence of 7–15% 26 affects antibiotics that can be prescribed. Remoteness and culture have maintained a very low rate of sexually transmitted infections (STI) for syphilis and HIV 27.

For this primary evaluation of diagnosis and management of UTI, all microbiological data of first episodes of non‐pregnant patients, 18 years and older, who attended an outpatient department from November 2013 to October 2014 with symptoms suggestive of a UTI (at least one of dysuria, frequency or flank pain), were reviewed.

### Clinical assessment and sample collection

In September and October 2013, healthcare workers were provided with a refresher course on urine samples. This included information on how to inform patients to obtain a clean MSU sample and for staff to accurately read a urine dipstick and handle and store a sample on site, prior to transportation for culture at the central laboratory of SMRU in Mae Sot, 30–60 km from the field sites. Information was made available in local languages to standardise the diagnostic procedures at all sites.

A single MSU sample was taken for all patients. The same sample was analysed using a rapid urine dipstick and urine microscopy in the field laboratory and stored in the fridge at 2–8 °C and transported to Mae Sot for urine culture. The speed of transportation depended on the day of the week with more than 85% of samples being collected and plated on the same day and the remainder being completed within two days. Data on demographics, clinical features, history of antibiotic use (last 14 days), diagnosis, initial management, dipstick, microscopy and culture results were extracted from the microbiology database.

### Laboratory procedures

ROCHE Combur‐10‐test UV/M^®^ dipstick tests were performed following the manufacturer's instructions. In brief, they were immersed in urine, laid flat and read after 60 s by a healthcare worker. Data for pH, protein, red blood cells (RBC), haemoglobin, ketones, leucocyte esterase and nitrites were available.

Urine microscopy was undertaken by trained staff in the field laboratory using a Hettick bench centrifuge EBA model 20. A urine volume of 10 ml was centrifuged at 800 ***g*** for 5 min. After removal of the supernatant, a drop of the mixed sediment was applied to a glass slide followed by a cover slip and was examined using light microscopy for 10 high‐powered fields (HPF; ×40 objective). The total counts of white blood cells (WBC), RBC, bacteria, epithelial cells, crystals (uric acid, oxalate and phosphate), casts (granular, RBC), yeast and trichomonas were divided by 10 to obtain the average per HPF. Bacteria were categorised as 1 +  (1–10 bacteria/HPF), 2 +  (11–100 bacteria/HPF) or 3 +  (>100 bacteria/HPF) 28. An open comment box was available for additional findings. The test was considered positive if WBC ≥10/HPF and bacteria ≥1/HPF, and a contaminated sample was classified as epithelial cells ≥5/HPF. In case of a contaminated sample, MSU was recollected for urine microscopy, dipstick and culture, if possible.

Urine culture was undertaken at the central microbiological facility in Mae Sot by trained staff. A sterile loop was used to inoculate 0.001 ml urine onto UTI chromogenic media (Oxoid Brilliance^™^ UTI Clarity^™^ agar, Basingstoke, Hampshire, United Kingdom) and incubated overnight in air at 35 ± 2 °C. The number of colony‐forming units (CFU) of each colony type was counted and categorised as follows: <10^4^, 10^4^–10^5^ and >10^5^ CFU/ml. Significant growth was classified as growth of a single organism at >10^5 ^CFU/ml. No significant growth was classified as a single organisms at <10^4 ^CFU/ml or two organisms at <10^5^ CFU/ml. Cultures growing three or more organisms were reported as mixed growth of greater than two organisms. For the analysis of POCT growth of a single organism at 10^4^–10^5^ CFU/ml, mixed growth of two organisms with one or both organisms at >10^5^ CFU/ml and known contaminants were excluded for analysis. All significant organisms were identified and antimicrobial susceptibility testing was performed, where appropriate, using standard operating procedures. ESBL testing was undertaken for *E. coli* and *Klebsiella* sp. isolates. Results were recorded and reported, using the in‐house Microbiology Microsoft Access 2010 database.

### Diagnosis

In practice, healthcare workers utilised the 2007 Burmese Border Guidelines for patients presenting with urinary tract symptoms 29. This guideline, adapted from the Médecins Sans Frontières guidelines, recommends the use of history taking, physical examination and POCT to diagnose patients with urinary tract symptoms without fever as urinary tract infection and with fever and flank pain as pyelonephritis.

### Treatment

The antibiotic repertoire for treatment of UTI in the field clinics is limited 30. For females, treatment for uncomplicated UTI is oral nitrofurantoin 100 mg QID for 3 days; for males, it is oral ciprofloxacin 500 mg BID for 7 days. G6PD status is checked before giving nitrofurantoin. Patients diagnosed with pyelonephritis are admitted and given oral ciprofloxacin 500 mg BID or IV ceftriaxone 50 mg/kg OD if oral intake is intolerable.

### Follow‐up

Routinely, if the patient was admitted, antibiotics were changed according to the organism and the susceptibility profiles. This information was normally available 48–72 h post‐sample receipt. No consistent routine follow‐up was arranged for outpatients, unless an ESBL‐producing bacterium or a bacterium resistant to the prescribed antibiotic was isolated. In both cases, an attempt to contact the patient either through phone calls or through a home visit was made in order to change the antibiotics. This was not always possible due to difficulties in knowing the exact address or living in areas that were difficult or unsafe to reach. All patients were advised to come back if their symptoms were not improving.

### Statistical analysis

The analysis was undertaken using IBM SPSS Statistics 22 for Windows. The mean was calculated for continuous normally distributed data and the median for non‐normally distributed data. Two‐way tables were used to obtain sensitivity, specificity, positive predictive and negative predictive values for different diagnostic approaches and cut‐offs. The diagnostics odds ratio (DOR) that represents the ratio of the odds of a positive test result in the diseased group, i.e significant culture, to the odds of a positive test in the non‐diseased group, i.e negative or non‐significant culture, was calculated using the following formula: 16

$$\text{DOR}=\frac{sensitivity/(1-sensitivity)}{\left(1-specificity)/(specificity\right)}$$

The specimens with contaminated culture and/or urine microscopy were reported but excluded from analysis.

### Ethics

Retrospective review of outpatient records was approved by the Oxford Tropical Research Ethics Committee and by the local Tak Province Community Ethics Advisory Board. All data were anonymised.

## Results

### General demographics

In one year, November 2013 to October 2014, records were reviewed for 247 non‐pregnant patients, 18 years or above with urinary tract symptoms. The median (range) age was 34 (18–78) years, and 82.6% (204/247) of patients were female. When available (data missing for 40 (16.2%)), the duration of symptoms was 3 days (1 day–2 years) with dysuria 81.2% (199/245), suprapubic pain 67.8% (166/245) and polyuria 53.7% (130/242) most commonly reported. There were 9.3% (4/43) of male patients who complained of penile discharge. Among these patients, 52.2% (128/245), reported a history of fever in the last 48 h of 2 (1–20) days duration, and 22.5% (54/240) of these had a documented fever ≥38 °C. There were 7.8% (19/245) of patients with a history of antibiotic intake in the last 2 weeks. The final working diagnoses given by healthcare workers were as follows: uncomplicated UTI 29.6% (73/247); pyelonephritis 27.5% (68/247); pyelolithiasis 4.0% (10/247); genito‐urinary infection 2.8% (7/247); unknown febrile illness 14.6% (36/247); and unable to provide a definitive diagnosis in 21.5% (53/247).

### Laboratory results

**Figure 1:**
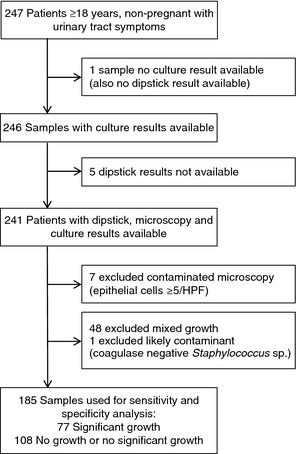
Samples for analysis of point‐of‐care tests and culture.

The samples included in analysis are described (Figure 1) and details of POCT are provided (Supporting Information) with the main findings as follows.

Results were not recorded for 2.4% (6/247) of the urine dipsticks. Positive nitrite and/or leucocyte esterase was observed in 76.8% (185/241) including 62.8% (27/43) of males; both leucocyte esterase and nitrite in 21.2% (51/241); positive nitrites with negative leucocytes in 3.3% (8/241); and proteinuria in 41.5% (100/241), haematuria in 32.0% (77/241) and ketones in 9.5% (23/241).

There were 7/247 (2.8%) samples with five or more epithelial cells which were excluded from further analysis due to contamination. There were 36.7% (88/240) of urine microscopy examinations that fulfilled the criteria for positivity (WBC ≥10, epithelial cells <5, bacteria ≥1), including 23.1% (9/39) of males. Crystals were present in 55.4% (133/240) of patients, most of whom had one crystal type reported 91.0% (121/133), with two and three types reported in 11 and one patient, respectively. The predominant crystal type was uric acid detected in 91.7% (122/133). RBC casts were present in one patient 0.4% (1/240), and WBC casts were present in two patients 0.8% (2/240). In the 197 female patients, 1.7% (4/240) had yeast and 0.4% (1/240) had trichomonas, but not identified in any males.

A urine culture result was not available for one patient. The greatest proportion of urine cultures, 45.1% (111/246), were found to have no significant growth or no growth at all; 32.9% (81/246) had significant growth (single organism ≥10^5^ CFU/ml), and 21.6% (53/246) had mixed growth or, in one case, 0.4% (1/246) a likely contaminant (coagulase negative *Staphylococcus* sp.). Half (50% (7/14)) of the patients reporting recent antibiotic use had a significant growth, and this accounted for 9.1% (7/77) of all positive cultures. Amongst the males, 18.6% (8/43) had a positive culture, all of which were in men younger than 50 years of age. Of the four men with penile discharge, one had a significant growth of *E. coli*.

There were 185 patients with valid POCT and urine culture, which are used in the remaining analysis (Figure 1). Isolated organisms in 41.6% (77/185) of significant cultures included the following: *E. coli* 87.0% (67), *Staphylococcus saprophyticus* 3.9% (3) and one (1.3%) each of *Klebsiella pneumonia*e, *Proteus mirabilis, Enterococcus* sp., *Burkholderia cepacia*,* Enterobacter cloacae*,* Enterobacter aerogenes* and *Salmonella* sp. Of the *E. coli* isolates, 20.9% (14/67) were ESBL producers. The one *Klebsiella pneumoniae* isolate was not an ESBL producer. Antibiotic susceptibilities for *E.coli* organisms are described in Table 1.

**Table 1:** Antimicrobial susceptibility of *E. coli* organisms in non‐pregnant adults on the Thailand–Myanmar border, 2013–2014 (*n* = 67)

	Resistant % (*n*)	Intermediate % (*n*)	Sensitive % (*n*)
Ampicillin	70.1 (49)	4.5 (3)	25.4 (17)
Ceftriaxone	20.9 (14)	0 (0)	79.1 (53)
Co‐amoxiclav	7.5 (5)	31.3 (21)	61.2 (41)
Cotrimoxazole	62.7 (42)	0 (0)	37.3 (25)
Ciprofloxacin	20.9 (14)	0 (0)	79.1 (53)
Gentamicin	20.9 (14)	0 (0)	79.1 (53)
Nitrofurantoin	0 (0)	0 (0)	100 (67)
Meropenem	0 (0)	0 (0)	100 (67)

In these 185 samples, every dipstick was positive if the microscopy was positive. This is apparent in the result of the DOR of leucocyte esterase ≥1 and/or nitrite positive and the DOR of urine microscopy and/or dipstick positive which are equal (Table 2). The highest DOR was obtained for positive dipstick combined with positive microscopy.

**Table 2:** Sensitivity and specificity of point‐of‐care tests to urine culture results in non‐pregnant adults

		*n*	Sensitivity % (*n*)	Specificity % (*n*)	PPV % (*n*)	NPV % (*n*)	DOR
Leucocyte esterase ≥1	All patients	185	94 (72/77)	49 (53/108)	57 (72/127)	91 (53/58)	13.88
	UTI^a^ diagnosis	97	94 (65/69)	11 (3/28)	72 (65/90)	43 (3/7)	1.95
Nitrite positive	All patients	185	48 (37/77)	94 (101/108)	84 (37/44)	72 (101/141)	13.35
	UTI^a^ diagnosis	97	52 (36/69)	79 (22/28)	73 (36/42)	4 (22/55)	4.00
Leucocyte esterase ≥1 ± nitrite positive	All patients	185	99 (76/77)	47 (51/108)	57 (76/133)	98 (51/52)	68.00
	UTI^a^ diagnosis	97	100 (69/69)	7 (2/28)	73 (69/95)	100 (2/2)	n/a
Blood ≥1 and protein ≥1	All patients	185	30 (23/77)	90 (97/108)	68 (23/34)	64 (97/151)	3.76
	UTI^a^ diagnosis	97	32 (22/69)	93 (26/28)	92 (22/24)	36 (26/73)	6.09
Microscopy WBC ≥10/HPF, epithelial cells <5/HPF, bacteria ≥1/HPF	All patients	185	57 (44/77)	89 (96/108)	79 (44/56)	74 (96/129)	10.67
	UTI^a^ diagnosis	97	64 (44/69)	57 (16/28)	79 (44/56)	39 (16/41)	2.35
Microscopy and/or dipstick positive	All patients	185	99 (76/77)	47 (51/108)	57 (76/133)	98 (51/52)	68.00
	UTI^a^ diagnosis	97	100 (69/69)	7 (2/28)	73 (69/95)	100 (2/2)	n/a
Microscopy and dipstick positive	All patients	109	98 (44/45)	81 (52/64)	79 (44/56)	98 (52/53)	190.67
	UTI^a^ diagnosis	58	100 (44/44)	14 (2/14)	79 (44/56)	100 (2/2)	n/a

PPV, positive predictive value, NPV, negative predictive value, DOR, diagnostic odds ratio which is not available (n/a) when sensitivity = 100.

a Includes diagnosis of UTI and pyelonephritis.

### Diagnosis

When the final diagnosis was an infection of the urinary tract, sensitivity of the field tests increased but specificity and the DOR decreased (Table 2). To elucidate this, further analysis (specificity; sensitivity; DOR) was calculated for the most commonly reported symptoms: dysuria (82%; 22%; 1.25), polyuria (58%; 55%; 1.71) and suprapubic pain (69%; 31%; 0.98). These low figures explain why the DOR figure decreases when healthcare workers make the diagnosis of UTI/pyelonephritis (Table 2).

### Treatment

**Figure 2:**
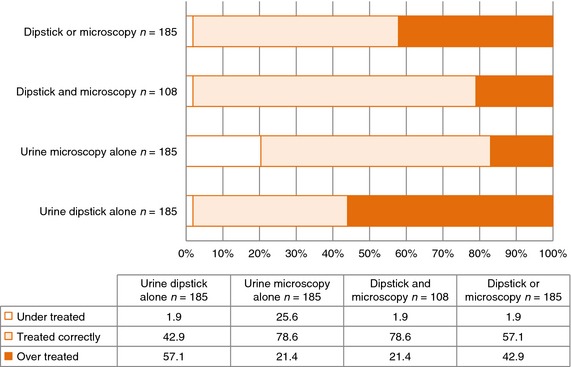
Hypothetical proportions (%) of over and under treatment based on retrospective analysis of point‐of‐care testing compared to urine culture.

Using only POCT to guide treatment (Figure 2), urine dipstick alone results in over treatment of 57.1% of patients, and urine microscopy alone results in under treatment of 25.6% of patients. Discrepant dipstick and microscopy results were associated with lower specificity, whilst the proportion of both under and over treatment are minimised by the combined use of dipstick and microscopy (Figure 2).

Healthcare workers prescribed antibiotics in 63.2% (156/241) of all patients. When both microscopy and urine dipstick results were positive they adhered to guidelines and prescribed correct antibiotics for 100% (85/85) of these patients. More than half, 56% (56/100) of the patients with discordant results (between microscopy and urine dipstick) received antibiotics. For negative microscopy and negative dipstick, 21.4% (12/56) received antibiotics, including doxycycline in eight for unknown fever, and 3.6% (2/56) were still diagnosed with a UTI.

Amongst 52.4% (97/185) of patients diagnosed with UTI or pyelonephritis, 71.1% (69/97) had a significant growth on culture, of whom 63.3% (31/49) had UTI and a significant growth and 79.2% (38/48) had pyelonephritis and a significant growth. There were 10.6% (7/66) of cases prescribed antimicrobials for which the target organisms were not fully sensitive (Table 3). Amongst the 47.6% (88/185) not diagnosed with UTI or pyelonephritis, there were 9.1% (8/88) who had a significant growth on culture and these were discharged without antibiotic treatment.

**Table 3:** Antimicrobial susceptibility of isolated organisms compared to prescribed antimicrobial in non‐pregnant adults

Antimicrobial prescribed^a^	Antimicrobial susceptibility		
	Resistant % (*n*)	Intermediate % (*n*)	Sensitive % (*n*)
Ciprofloxacin *n* = 32	9.4 (3)	0 (0)	90.6 (29)
Ceftriaxone *n* = 7	28.6 (2)	0 (0)	71.4 (5)
Nitrofurantoin *n* = 27	3.4 (1)	3.4 (1)	86.2 (25)

a Total is 66 (not 69 as reported earlier in results) as one sample report failed to include antibiotic susceptibility, and in two samples, susceptibilities were not undertaken for Burkholderia cepacia and Salmonella sp. according to standard operating procedures.

## Discussion

Not unexpectedly, this rural and remote population had many of the typical features reported previously on community‐acquired UTI including a preponderance of female patients 5, a high proportion of infection due to *E. coli* 31 and discordance between POCT and gold standard urine culture 14, 16, 32. This analysis demonstrates that the use of positive dipstick (positive leucocyte esterase and/or nitrite) alone in patients presenting with urinary symptoms in this population will result in a high rate of over treatment (57.1%). In contrast, positive microscopy (WBC ≥10/HPF, bacteria ≥1/HPF) alone will result in under treatment (25.6%). In this setting, a combination of these modalities results in a logical methodological approach for diagnosis of UTI.

Obtaining both high sensitivity and specificity for diagnosis of UTI is not possible with current POCT 14. The results suggest that continuing to test all patients with both microscopy and dipstick and not treating those when both are negative, treating those when both are positive and observing those with discordant results will limit unnecessary antibiotic use and introduce the concept of antibiotic stewardship. In addition, continuing urine microscopy in this setting has a role in identifying acute kidney injury, for example in acute tubular necrosis, the urine typically contains tubular epithelial cells and cell casts of tubular epithelium 33. Urine microscopy is a more complex procedure than urine dipstick, requiring laboratory facilities, trained staff and quality control. Where microscopy for malaria and TB diagnosis is established, continuing use of this POCT is cost‐effective and it is appropriate to strengthen techniques and encourages reproducibility of results.

High levels of resistant organisms were cultured (20.9% of *E. coli* produced ESBL) although this is comparatively lower than the 37% reported from 97 samples (2009–2010) from two Thai hospital (Sirriraj Hospital in Bangkok and Songklanakarin Hospital, Songkhla, Southern Thailand) described in the Study for Monitoring Antimicrobial Resistance Trends (SMART) 34. It is, however, five times higher than described in the same setting in adult febrile pregnant women just 8 years earlier (2004–2006) 22. In addition, rates of antibiotic resistance against cephalosporins, ampicillin, cotrimoxazole and gentamicin were comparable to *E. coli* susceptibilities of community‐acquired infections in Asia 35. In view of this high and increasing resistance pattern, single‐dose fosfomycin may be beneficial in this setting 36.

This analysis quantified the proportion (10.6%) of patients who received, per guideline, antimicrobials to which the organism was not fully susceptible. Staff education to specifically inform patients about this possibility is ongoing. Despite more than half of the patients being able to provide a mobile phone contact number, this remains an unreliable method of contact due to economics (the number expires with the special deal), remoteness and the mobile nature of the migrants.

At SMRU, the diagnosis by healthcare workers was accurate when both POCT were positive but specificity decreased with discordant results and inclusion of clinical judgement (Table 2). This may, in part, be explained by language difficulties. Whilst Paw Karen, Sgaw Karen and Burmese are the languages directly used for patient care, medical language is more frequently translated from English. Hence, the healthcare worker is at the intersection of translating patient symptoms to medical terms and correlating the terms in Karen or Burmese to English. As an example, dysuria, the most common symptom, often translated as ‘burning urine’ is mistakenly assigned to patients with fever who have the sensation of hot urine. This distinction needs to be emphasised. Medical education in developed countries puts a strong emphasis on clinical history for diagnosis, yet healthcare workers in resource poor settings have usually not had this educational opportunity and are more reliant on POCT 37.

## Limitations

Recent antibiotic use may have been underestimated as it is lower than the 25% reported in a 2011 survey in women from the same area. STI as a cause of urinary symptoms and false‐positive POCT cannot be excluded, but only a small number of men complained of penile discharge, and a low incidence of sexually transmitted infections is reported in pregnant women 27. This work was undertaken in a low resource setting with healthcare workers without professional qualifications and therefore may not be applicable in a developed setting.

## Conclusion

The use of microscopy and dipstick concurrently is beneficial for improved diagnosis of UTI when they are concordant, allowing appropriate prescribing of antibiotics. Discordant results remain problematic but are a target area for antibiotic stewardship. Microscopy requires training and quality control but does permit a range of additional diagnoses for which more sophisticated testing remains lacking in many rural areas.

## Supporting information


**Table S1** Urine dipstick (ROCHE Combur‐10‐test UV/M® dipstick tests).
**Table S2** Urine microscopy results examined under 10 high powered fields (HPF; x40 objective).
