# Supplementary material for: The role of point‐of‐care tests in antibiotic stewardship for urinary tract infections in a resource‐limited setting on the Thailand–Myanmar border
Source: Trop Med Int Health. 2015 Jun 11;20(10):1281–9. doi: 10.1111/tmi.12541 (PMC4758398; doi:10.1111/tmi.12541)
Supplement: Supplementary file 1 — Table S1 Urine dipstick (ROCHE Combur‐10‐test UV/M® dipstick tests). Table S2 Urine microscopy results examined under 10 high powered fields (HPF; x40 objective). [file TMI-20-1281-s001.docx]

**Supplementary Information**

**Table 1 Urine dipstick (ROCHE Combur-10-test UV/M® dipstick tests)**

| **Characteristics** | **Result** | **n=241** | **Proportion** |
| --- | --- | --- | --- |
| **Leukocyte Esterase (LE)** | neg | 64 | 26.6 |
|  | 1+ | 49 | 20.3 |
|  | 2+ | 56 | 23.2 |
|  | 3+ | 72 | 29.9 |
| **Nitrites** | pos | 59 | 24.5 |
|  | neg | 182 | 75.5 |
| **LE &/or Nitrites** | pos | 185 |  |
| **LE & Nitrites (Nit)** | LE pos Nit pos | 51 | 21.2 |
|  | LE pos Nit neg | 126 | 52.3 |
|  | LE neg Nit pos | 8 | 3.3 |
|  | LE neg Nit neg | 56 | 23.2 |
| **pH** | 5 | 45 | 18.7 |
|  | 6 | 73 | 30.3 |
|  | 7 | 66 | 27.4 |
|  | 8 | 39 | 16.2 |
|  | 9 | 18 | 7.5 |
| **Protein** | neg | 141 | 58.5 |
|  | 1+ | 67 | 27.8 |
|  | 2+ | 25 | 10.4 |
|  | 3+ | 8 | 3.3 |
| **Erythrocytes^a^** | pos | 77 | 32.0 |
|  | neg | 164 | 68.0 |
| **Hb ^a^** | pos | 65 | 27.0 |
|  | neg | 176 | 73.0 |
| **Ketones** | neg | 218 | 90.5 |
|  | 1+ | 11 | 4.6 |
|  | 2+ | 10 | 4.1 |
|  | 3+ | 2 | 0.8 |
| **Glucose** | neg | 236 | 97.9 |
|  | > 1+ | 5 | 2.1 |
| ^a^ 16 patients had both erythrocytes and Hb present | | | |

**Table 2 Urine microscopy results examined under 10 high powered fields (HPF; x40 objective).**

| **Characteristiscs** | **Result** | **n=247** | **Proportion** |
| --- | --- | --- | --- |
| **WBC** | 0 to <5 | 132 | 53.4 |
|  | 5 to <10 | 12 | 4.9 |
|  | equal or less 10 | 103 | 41.7 |
| **Epithelial** | Clean <5 | 240 | 97.2 |
|  | Dirty 5 or more | 7 | 2.8 |
| **RBC** | 0 to <2 | 185 | 74.9 |
|  | 2 to <5 | 25 | 10.1 |
|  | 5 to 10 | 19 | 7.7 |
|  | more than 10 | 18 | 7.3 |
| **Bacteria** | 0 | 13 | 5.3 |
|  | 1+ | 172 | 69.6 |
|  | 2+ | 41 | 16.6 |
|  | 3+ | 21 | 8.5 |
| **Crystals** | Absent | 113 | 54.3 |
|  | Present | 134 | 45.7 |
|  | Uric Acid | 123 | 91.8 |
|  | Oxalate | 14 | 5.7 |
|  | Phosphate | 11 | 4.5 |
| **Casts** | Granular casts | 0 | 0.0 |
|  | RBC Casts | 1 | 0.4 |
| **Other** | Candida | 4 | 1.6 |
|  | Trichomonas | 1 | 0.4 |
